# Case Reported

**Published:** 1876-05

**Authors:** D. C. Wilson

**Affiliations:** Ironton, Ohio


### CASE REPORTED BY D. C. WILSON, M.D.,

OF IRONTON, OHIO.

On November 27th, 1875, at 5 o'clock, p.m., I was called to see a child aged two years. On examination I found it anemic: the glands of the neck, on the left side, were very much enlarged, and quite hard. The left foot, and left leg, half-way to the knee, were swollen, and of a black color, having all the appearance of gangrene. The foot and leg were cold, but did not pit on pressure; there was no fever. pulse natural, tongue clear, bowels regular, and appetite good. There was also a slight purplish discoloration on the inside of each thigh.

The mother said, about an hour before sending for me the child was as well as usual, and had been sleeping, when, on awakening, she noticed this discoloration on its foot and leg, when she became alarmed and sent for me. She also stated that sometime ago she had consulted a doctor in regard to the glandular enlargements, and he had treated the child; but said it was well, and it had not taken any medicine for two weeks prior to consulting me. I ordered tonics and stimulants internally, and the application ot warm cloths to the extremities.

Nov, 28, 3 p.m. Patient rested well all night; was sitting on its mother's lap eating bread when I called. The discoloration had entirely disappeared from the left foot and leg, and both thighs, but had made its appearance on the right leg, just above the ankle posterior, to the extent of two by four inches, and was very black. It also had made its appearance on the right side of the face and neck, covering almost this entire region. Same treatment continued.

Nov, 29, 9 a.m. Patient has been quite restless all night; refuses to eat this morning. The discoloration has extended over the entire surface of both legs and feet, almost to the body. There is also a large black place, about six inches square, on the right gluteal region; child very weak and feeble, pulse irregular and weak, extremities cold. I ordered stimulants every half-hour, in the hope I might bring on reaction, as I saw the child was rapidly failing, but my efforts were futile; the patient continued to fail, and expired at 4 o'clock, p.m., not living quite forty-eight hours.

The interesting features of this case, I think, was the rapid disappearance and re-appearance of the discoloration, and the rapidity with which the disease produced death, during the course of a tonic and stimulating treatment.

